# Undergraduate medical students’ attitudes towards medical errors and patient safety: a multi-center cross-sectional study in the Gaza Strip, Palestine

**DOI:** 10.1186/s12909-020-02375-z

**Published:** 2020-11-19

**Authors:** Mohammed Alser, Bettina Böttcher, Maha Alfaqawi, Abdallah Jlambo, Walaa Abuzubaida, Nasser Abu-El-Noor

**Affiliations:** 1 Faculty of Medicine, Islamic University of Gaza, PO Box 108, Remal, Gaza, Gaza Strip Palestine; 2 Nasser Medical Complex, Palestinian Ministry of Health, Khan Younis, Gaza Strip, Palestine; 3 Faculty of Medicine, Al-Azhar University, Gaza Strip, Palestine

**Keywords:** Patient safety attitudes, Medical students, Undergraduate medical education, Understanding of medical error, Gaza Strip, Palestine

## Abstract

**Background:**

In undergraduate medical education, patient safety concepts and understanding of medical errors are under-represented. This problem is more evident in low-income settings. The aim of this study was to explore undergraduate medical students’ attitudes towards patient safety in the low-income setting of the Gaza Strip.

**Methods:**

A cross-sectional, descriptive study included medical students of the two medical schools in the Gaza Strip with 338 medical students completing the Attitudes to Patient Safety Questionnaire-IV (APSQ-IV), which examines patient attitudes in 29 items over 10 domains. Results are represented as means ± standard deviations for each item and domain as well as percentage of positive responses to specific items.

**Results:**

Medical students reported slightly positive patient safety attitudes (4.7 ± 0.5 of 7) with the most positive attitudes in the domains of situational awareness, importance of patient safety in the curriculum, error inevitability and team functioning. While no negative attitudes were reported, neutral attitudes were found in the domains of professional incompetence as a cause of error and error reporting confidence. Study year and gender had no significant association with patient safety attitudes, except for disclosure responsibility, where male students displayed significantly more positive attitudes. The study university was significantly associated with three of the 10 examined domains, all of which involved understanding of medical errors, for which students of University 2 (who had undergone limited patient safety training) held significantly more positive attitudes, compared with students of University 1 (who did not have structured patient safety training).

**Conclusion:**

Medical students’ patient safety attitudes were very similar among students from both universities, except for understanding of medical error, for which students, who had received structured training in this topic, displayed significantly more positive attitudes. This underlines the power of the ‘hidden curriculum’, where students adjust to prevalent cultures in local hospitals, while they do their clinical training. Furthermore, it highlights the need for a systematic inclusion of patient safety content in local undergraduate curricula.

## Strengths and limitations of this study

- The sample in this study included a large proportion (64.6%) of the target population from both universities and can, therefore, be regarded as representative for medical students in the Gaza Strip.
- The research tool has been devised specifically for medical students and showed good reliability in the Arabic translation. However, this translation has not yet been formally validated.
- The study describes the attitudes of students but did not examine actual behaviour, which is more important for the improvement of patient outcomes but has to be investigated by a separate study design.

## Background

The World Health Organization estimated that 1 in 10 patients is harmed while receiving hospital care in high-income countries and 1 in 300 will die as a result [1–3]. Moreover, 50% of such adverse events, leading to patient harm, were considered to be preventable [4–6]. In low- and middle-income countries, harm to patients was found to occur in 8% of hospital admissions with 83% of them deemed to be preventable [7, 8]. In Palestine, as many as 1 in 7 patients admitted to hospitals were found to come to harm with 59.3% of incidents thought to be preventable [9]. Therefore, improving patient safety has become a priority and a cornerstone to improving the delivered quality of care. Patient safety contents have been included in postgraduate and undergraduate education of health-related professions worldwide [10–12]. Furthermore, it was shown that learning about patient safety early in the career and at the undergraduate level is more effective than later on at the postgraduate level [13, 14]. One reason could be the ‘hidden curriculum’, where medical students and young doctors learn from role models or more senior doctors who might demonstrate clinical practice that does not always adhere to patient safety standards [15, 16]. Different studies showed conflicting results in the impact of study year on patient safety attitudes, where some found students in earlier years demonstrating more positive patient safety attitudes and others, mainly US and UK based studies, found final year students with more positive attitudes [13, 17–20]. This difference could be driven either by more positive patient safety attitudes prevalent in the health systems of high-income countries, by more systematic integration of patient safety content in undergraduate curricula than currently is the case in low- and middle-income countries or by a combination of both these factors [21–24]. Therefore, it is essential for students to enter their work life as doctors with a firm and solid foundation in patient safety knowledge, skills and attitudes, as this might create a pathway to improve patient safety practices and, thus, provide better quality services. In order to achieve this, medical students have to experience a combination of formal teaching about patient safety concepts, as well as organisational culture in their clinical learning environments, that reflect the formal learning they received. So far undergraduate patient safety interventions have been effective in producing improvement in patient safety attitudes, as well as self-reported behaviours, but better patient outcomes as a result of patient safety interventions at an undergraduate level still have to be proven [21–24].

Some patient safety concepts, such as the importance of teamwork and communication, benefitted from wide global exposure, also within specific emergency training courses. Other concepts have been less prominent, such as involving patients in their care, which remains a challenge in many settings [5, 25–27]. Furthermore, concepts, such as the systems approach to ensure patient safety, error causation in medicine and the importance of error reporting contribute to the improvement of patient safety understanding [10, 28–32]. These concepts emphasize the crucial role good systems with well-designed policies and protocols play in the avoidance of errors in medicine [10]. However, equally important is learning from adverse events on an individual as well as organisational level to avoid similar events in the future [30–32]. In order to learn from adverse events, an organization has to foster transparency within the organization and beyond. For this, disclosure of errors (or potential errors) is important and all healthcare workers should be involved in this process [5, 10, 30–32]. Disclosure of errors can be challenging, especially in a culture, where blame for adverse events is commonly attributed to individual practitioners, such as in the setting of this study. Healthcare staff at undergraduate as well as postgraduate levels can be intimidated by fear of disciplinary action or a loss of face resulting from disclosure of errors. This is especially prevalent in settings where admitting to errors is regarded as a weakness, such as in the Arab context [33–35]. An incidence reporting system has to be provided within healthcare organisations, which is presently not available in the local context. Furthermore, timely feedback to staff about actions taken as a result of such reported incidences will be necessary to enhance staff engagement and trust in such a system [30, 36–39]. ‘Disclosure training’ proved to be useful in organisations, both to motivate staff to report incidences and to develop an organisational culture to learn from them [38–40]. In settings, where patient safety contents have not been integrated in the undergraduate or postgraduate curricula, error disclosure, understanding errors and learning from errors can be more challenging [21, 25, 26]. Hence, inclusion of such contents in the undergraduate curriculum remains a pressing issue in many areas.

Patient safety attitudes influence patient outcomes, with positive attitudes being linked to more positive patient outcomes and vice versa [41, 42]. Patient safety outcomes were found to be moderately positive among healthcare providers in Palestine [25, 43–45], despite a paucity of structured patient safety training. So far, patient safety content has not been represented well in post- and undergraduate training of healthcare professionals in low- and middle-income countries [21], and Palestine is no different. Similarly, only one local university introduced limited patient safety contents into its curriculum, although patient safety education might have greater impact in undergraduate education than in postgraduate education [13, 14].

Little is known about medical students’ attitudes toward patient safety in Palestine, including the Gaza Strip, although it is a crucial time for them to be introduced to patient safety concepts. Therefore, this study assessed the attitudes of medical students toward patient safety at the two universities with medical faculties in the Gaza Strip.

## Methods

### Study design, study setting and study population

This was a descriptive, cross-sectional study. The target population was medical students at the only two universities in the Gaza Strip that offer medical education. The study included students in Years 4, 5 and 6, as these students are receiving clinical training. Students in Years 1, 2 and 3 were excluded, as they do not have any clinical experience yet.

### Sampling process

A census sample was used and questionnaires were delivered by hand (by members of the research team who are not involved in teaching at any of the two universities) to eligible medical students at the two faculties of medicine in both universities at the end of their lectures and clinical teaching sessions. All 523 students enrolled in Years 4, 5 and 6 at both universities during the study period were eligible and were invited to participate in the study. Prior to completion of the questionnaires, the purpose of the study was explained to potential participants. It was also emphasized that participation was voluntary and would not impact on their academic performance. Furthermore, the confidentiality of all questionnaires was assured. In total, 338 (64.6%) students returned their completed questionnaires to the research team.

### Research instrument

The instrument used in this study was the Attitudes to Patient Safety Questionnaire IV (APSQ-IV). This version is a modified version of the APSQ-III [46]. A permission to translate and use the instrument was obtained from the authors. This tool examines patient safety attitudes over 10 domains using 29 individual items that are rated on a 7-point Likert scale, ranging from 1 (very strongly disagree) to 7 (very strongly agree). The examined domains included general patient safety attitudes (2 items), patient safety training received (3 items), error reporting confidence (3 items), error inevitability (4 items), professional incompetence as an error cause (2 items), disclosure responsibility (3 items), team functioning (3 items), patient involvement to reduce error (3 items), importance of patient safety training (3 items) and situational judgement (3 items). A higher score indicated a more positive response to the item concerned. This instrument was designed to assess the attitudes of medical students toward safety culture [46]. Nine items of the instrument were negatively worded and scores were reversed and recoded before data analysis.

The APSQ-IV was translated into the Arabic language by two bilingual members of the research team who have significant experience in health research and survey design [47]. Another two bilingual healthcare professionals back-translated the research tool into English to ensure consistency. Following this, face validity was assessed by seven doctors working in clinical practice for at least 6 years (range: 6–14 years), who reviewed the translation. The reviewers provided a few suggestions to improve the quality of the translation and to make it more user-friendly and comprehensive. The final version of the tool was modified accordingly and then pretested on 10 medical students from the involved universities. These 10 medical students were excluded from the study.

### Missing values

The total number of missing values was 97, representing 0.9% of 9802; the total number of examined items. This total number of examined items was calculated by multiplying the number of items per questionnaire (29) by the number of participants (338). The missing values were distributed randomly across items, ranging from 0 to 6 missing values per item (0–1.7%), Missing data were replaced by serial means for each variable.

### Data analysis

Each participant’s response was summed up into ten sub-scores that corresponded to the ten key domains. The Total APSQ-Scores were calculated by adding the response to all items for each participant. The Overall APSQ-Score describes the overall patient safety attitudes as a Likert-scale score with a maximum of 7. This was calculated by dividing each Total APSQ-Score by the total number of items and then representing this score as one mean (±standard deviation) for all participants. Furthermore, the proportion of participants giving a positive score on a single item (defined as a score of 5, 6 or 7) is represented for each item as the percentage of positive responses from all responses. Cronbach’s Alpha was used to test for reliability of the questionnaire, which was 0.766, indicating good reliability.

One-way ANOVA and t-test were used to examine any association between participants’ characteristics and overall APSQ scores. A *p*-value of ≤0.05 was considered statistically significant. All statistical analyses were performed using the Statistical Package for the Social Sciences (SPSS) for Windows version 23.

### Ethical considerations

Approval for the present study was obtained from the ethics commission of one university as well as the administration of both universities. The purpose of the study was fully explained to all participants, all data were collected and kept completely anonymously and written consent had been obtained from all participants by a research team member prior to completing the questionnaire. Participants were informed that their participation was entirely voluntary and their decision to participate or not had no influence on their academic performance.

## Results

A total of 338 students from the only two medical faculties in the Gaza Strip participated in this study with a response rate of 64.6% (338/523) of all clinical students in these faculties during the study period. The majority of participants were males (*n* = 200; 59.2%) and from Year 5 (*n* = 158; 46.7%). University 2 was represented by 188 participants (55.6%, Table 1). Out of all participants, 38 students (11.2%) stated that they had made at least one medical error during their clinical training, 24 (63.2%) of them had told their clinical supervisors about committing this error. However, 130 students (38.5%) reported having seen another colleague committing a medical error (Table 1) and 37 of them (28.5%) reported this to their supervisors.

**Table 1:** General Characteristics of the respondents

Variable	Number (%) (***n*** = 338)	Overall APSQ score Mean (±SD)	*p*-value
**Gender**			
Males	200 (59.2)	4.8 (0.53)	0.11
Females	128 (40,8)	4.7 (0.48)	
**Study Year**			
4	95 (28,1)	4.7 (0.57)	
5	158 (46.7)	4.8 (0.50)	0.14
6	85 (25.1)	4.7 (0.47)	
**University**			
1	150 (44,4)	4.7 (0.53)	
2	188 (55.6)	4.8 (0.51)	0.42
**Students reported having made a medical error during their clinical training**			
Yes	38 (11.2)	5.0 (0.44)	0.04
No	300 (88.8)	4.7 (0.52)	
**If yes: Students who told their supervisor about it**			
Yes	24 (63.2)	5.0 (0.42)	
No	14 (36.8)		
**Students who reported witnessing a colleague (doctor or student) do a medical error during their clinical training**			
Yes	130 (38.5)		
No	208 (61.5)		
**If yes: Students who told their supervisor about this**			
Yes	37 (28.5)		
No	93 (71.5%)		

### Attitudes of medical students toward patient safety

The mean overall score of medical students’ attitudes toward patient safety was 4.7 (±0.5 SD) from the maximum score of 7 (Table 2). The ‘Situational awareness’-score was the highest score among the ten domain scores with a mean value of 5.6 (±1.1 SD), followed by “Error inevitability” and “Importance of patient safety in the curriculum” scores with 5.0 (±0.8 SD) and 5.0 (±0.9 SD) respectively (Table 2). On the other hand, the lowest score was collected for “Professional incompetence as a cause of error”, showing neutral attitudes with a mean score of 4.2 (±1.2 SD). No negative attitudes (mean scores < 4) were reported by the students (Table 2).

**Table 2:** Results for individual items in means ± standard deviation (SD) with a maximum score of 7 as well as the percentage of positive responses

Item	Mean (±SD)	% of positive responses
**S1_Patient safety general**	**4.7 (1.0)**	
Most harm to patients is unavoidable (R)	4.0 (1.6)	39.6
When things go wrong, learning from error is more important than disciplining individuals	5.3 (1.5)	70.7
**S2_Patient safety training received to date**	**4.5 (1.2)**	
My training is preparing me to understand the cause of errors	4.5 (1.5)	54.7
I have a good understanding of patient safety as a result of my training	4.8 (1.4)	68.6
My training is preparing me to prevent medical errors	4.2 (1.5)	46.7
**S3_Error reporting confidence**	**4.3 (1.3)**	
I would feel comfortable reporting any errors I had made no matter how serious the outcome had been for the patient	5.0 (4.1)	51.5
I would feel comfortable reporting any errors other people had made, no matter how serious the outcome had been for the patient	4.0 (1.7)	39.9
I am confident I could talk openly to my supervisor about an error I had made if it had resulted in potential or actual harm to my patient	5.0 (1.6)	57.7
**S4_Error _inevitability**	**5.0 (0.8)**	
Human error is inevitable	5.9 (1.6)	82
Very experienced health professionals make errors	5.8 (1.4)	85.2
The clinical environment can cause errors	5.7 (1.3)	82.5
If people paid more attention to work, medical errors would be avoided (R)	2.6 (1.4)	10.7
**S5_Professional incompetence as a cause of error**	**4.2 (1.2)**	
Medical errors are a sign of incompetence (R)	5.1 (1.6)	65.7
Most medical errors result from careless health professionals (R)	3.5 (1.6)	25.4
**S6_Disclosure responsibility**	**4.5 (1.2)**	
Doctors have a responsibility to disclose errors to patients only if they result in harm (R)	3.8 (1.8)	33.7
All medical errors should be reported	5.0 (1.6)	65.4
It is not necessary to report errors which do not result in harm for the patient (R)	4.8 (1.7)	59.2
**S7_Team functioning**	**4.9 (0.8)**	
For optimum safety cooperation, sharing of information is crucial	6.0 (1.4)	87.9
Junior members of a team should think carefully before speaking up about patient safety (R)	2.9 (1.7)	18.3
The safest teams are those, where different professional groups learn from and with each other	5.9 (1.3)	84.3
**S8_Patient role in error management**	**4.5 (0.8)**	
Patients have an important role in preventing medical errors	4.8 (1.5)	64.4
Actively seeking feedback from patients about quality and safety of care is important for patient safety.	5.5 (1.4)	77.8
Patients are not aware of how safe their care is (R)	3.2 (1.4)	17.8
**S9_Importance of patient safety in the curriculum**	**5.0 (0.9)**	
Teaching students about patient safety should be an important priority in training undergraduates	5.9 (1.4)	85.2
Learning about patient safety issues before I qualify will enable me to become a more effective doctor/nurse	5.5 (15)	79.3
Patient safety issues cannot be taught and can only be learned through clinical experience when qualified	3.5 (1.8)	31.7
**S10_Situational awareness**	**5.6 (1.1)**	
Being on the look-out for potential risks can be detrimental to patient safety (R).	4.7 (1.8)	57.4
Planning together to deal with problems that may arise is important for patient safety	5.9 (1.4)	85.5
Understanding the roles and responsibilities of every member of the team is important for patient safety	6.1 (1.3)	89.1
***Overall Score (max 7)***	**4.7 (0.5)**	

*(R)* Reversed scored item

In total, 11 individual questionnaire items had a positive response rate of > 70%, meaning that > 70% of participants scored 5, 6 or 7 on these items. The greatest proportion of participants with positive attitudes was found for the item “Understanding the roles and responsibilities of every member of the team is important for patient safety” with 89.1% followed by “For optimum safety cooperation, sharing of information is crucial” with 87.9% of students giving a positive response to this item. On the other hand, less than 40% of participants displayed positive attitudes towards eight items including two reversely scored items: “If people paid more attention at work, medical errors would be avoided (R)” with 10.7% and “Patients are not aware of how safe their care is (R)” with 17.8% of students (Table 2).

### Association between participants’ characteristics and APSQ scores

No statistically significant differences were found in the overall APSQ scores in relation to the students’ characteristics (university, study year and gender; Table 3). However, University 2 showed statistically significantly higher sub-scores within the domains of “Error reporting confidence”, “Error inevitability” and “Professional incompetence as a cause of error” (*p* ≤ 0.05). Furthermore, male participants displayed statistically significantly more positive attitudes in the domain of “Disclosure responsibility” (*p* ≤ 0.05; Table 3).

**Table 3:** Association between participants’ characteristics and APSQ scores, represented as mean scores out of the maximum of 7 ± standard deviaion (SD)

Item	University MEAN(±SD)			Study year MEAN(±SD)				Gender MEAN(±SD)		
	1	2	*P* value	4	5	6	*P* value	male	female	*P-*value
Patient safety general	4.7(1.0)	4.7(1.2)	0.89	4.6(1.0)	4.8(1.0)	4.6(1.1)	0.44	4.7(1.1)	4.6(0.9)	0.23
Patient safety training received to date	4.4(1.2)	4.5(1.2)	0.39	4.4(1.4)	4.6(1.1)	4.3(1.1)	0.16	4.5(1.2)	4.5(1.2)	0.71
Error reporting confidence	4.5(1.2)	5.1(0.8)	0.05	4.3(1.4)	4.4(1.3)	4.2(1.2)	0.53	4.4(1.3)	4.3(1.3)	0.75
Error inevitability	4.9(0.8)	5.1(0.8)	0.02	4.9(0.7)	5.0(0.8)	5.0(0.8)	0.65	5.0(0.8)	4.9(0.8)	0.16
Professional incompetence as a cause of error	4.1(1.1)	4.3(1.3)	0.04	4.2(1.2)	4.3(1.2)	4.2(1.3)	0.82	4.3(1.3)	4.1(1.1)	0.36
Disclosure responsibility	4.6(1.2)	4.5(1.1)	0.39	4.4(1.1)	4.5(1.1)	4.7(1.3)	0.33	4.6(1.2)	4.4(1.0)	0.05
Team functioning	4.9(0.8)	5.0(0.8)	0.20	4.9(0.8)	5.0(0.8)	4.9(0.9)	0.46	5.0(0.8)	4.9(0.8)	0.51
Patient role in error management	4.4(0.9)	4.6(0.8)	0.10	4.4(0.9)	4.6(0.8)	4.5(0.8)	0.39	4.6(0.9)	4.5(0.7)	0.40
Importance of patient safety in the curriculum	5.0(0.9)	5.0(0.8)	0.55	5.0(1.0)	5.0(0.9)	4.9(0.7)	0.31	5.0(0.9)	5.0(0.9)	0.79
Situational awareness	5.6(1.3)	5.6(1.0)	0.90	5.4(1.1)	5.6(1.2)	4.7(0.4)	0.27	5.6(1.1)	5.6(1.2)	0.80
**Total score**	4.7(0.5)	4.8(0.5)	0.42	4.7(0.5)	4.8(0.5)	4.7(04)	0.14	4.8(0.5)	4.7(0.4)	0.11

## Discussion

Medical students participating in this study demonstrated slightly positive attitudes toward patient safety. The most positive attitudes were found in the domains of ‘situational awareness’, ‘the importance of patient safety in the curriculum’, ‘error inevitability’ and ‘team functioning’. While no negative attitudes were reported by the students, neutral attitudes were found in the domains of professional incompetence as a cause of error and error reporting confidence. Study year and gender had no significant association with patient safety attitudes, except for disclosure responsibility, where males displayed significantly more positive attitudes than females. The University was only significantly associated with three of the 10 examined domains, all of which involved understanding of medical errors, for which students of University 2 held significantly more positive attitudes, compared with students of University 1.

In the Gaza Strip, two universities train medical students, and doctors have only been fully qualified from these universities within the last 10 years. Patient safety training has been included in the local curriculum only over the last 4 years and more systematically as a part of clinical skills courses, in one university (University 2). The clinical training of medical students is done at local hospitals and medical students of both universities have largely the same clinical teachers for this, making it safe to assume that they mostly share similar experiences during their clinical training.

Similar to healthcare professionals in the Gaza Strip, medical students demonstrated positive attitudes towards the domains of ‘Team functioning’ and ‘Inevitability of errors’, but almost neutral ones in ‘Error reporting confidence’ and ‘Professional incompetence as a cause of error’. [25, 33] Such similarities between healthcare professionals, working in hospitals and clinical students with very limited clinical experience, is very surprising and might be an example of the power of the ‘hidden curriculum’, where students learn attitudes from their role models and clinical teachers and from the culture in the organisations, where they receive their medical training [15–17, 48]. The majority of the current medical workforce in Gaza received their medical training in a plethora of different countries, mainly Arab countries (Egypt, Syria, Sudan, Algeria and Yemen), Western Europe (Germany), a number in Eastern European countries (Russia, Ukraine and Romania) and the United States of America (USA). Therefore, their exposure to patient safety contents in their undergraduate training might vary greatly and the majority had not received previous patient safety training [49]. Encouragingly, students found that the inclusion of patient safety contents in the curriculum was important, which was the domain with the most positive attitudes, compared to local healthcare professionals, who had their most negative scores in this domain [49]. This should alert medical educators to involve patient safety in their future curricula. Other studies also found medical students to be positive towards the inclusion of patient safety contents in the curricula [11, 50–53]. However, despite the importance they felt for inclusion of patient safety contents in the curricula, the majority of students found that patient safety was mainly learned in clinical practice, rather than in lecture rooms. This shows an awareness of the students of the impact of the ‘hidden curriculum’ and underlines the need for postgraduate training of healthcare professionals. Healthcare professionals with positive patient safety attitudes will be able to contribute positively to students’ and junior healthcare professionals’ understanding of patient safety and, thus, achieve improved patient outcomes [54].

In concordance with other studies, medical students in this study displayed poor understanding of error causation, as well as little error reporting confidence [11, 17–19, 48, 51, 55, 56]. Although students in most studies found that errors were inevitable and often demonstrated an understanding of ‘human factors’ in error causation, they displayed only little understanding of the role of systems in error causation and avoidance of error [13, 17, 20, 52]. The students’ attitudes to medical errors was less positive than those in other patient safety domains; similar to findings of other studies looking at self-reported patient safety competencies [48, 57]. In this study, most students perceived medical errors to be inevitable. But, at the same time, they found that most mistakes resulted from careless healthcare professionals. This paradox highlights the understanding of error to be that of an individual failing of a single healthcare professional, as students think that errors could be prevented if healthcare professionals took more care. Participants acknowledged that mistakes were inevitable, which is in accordance with the human factor concept, that in systems where humans act, mistakes might happen. However, they did not demonstrate an understanding that the impact of such errors could be alleviated by a systems-based approach throughout the healthcare system [28, 29]. Moreover, students did not appear to understand errors to be an opportunity to learn, as they felt reporting was not necessary, unless the patients had been harmed by these errors [56, 57]. This understanding is a reflection of the culture within the healthcare system, where the admission by healthcare professionals to not being certain on any point, may be regarded as a weakness [34, 35]. Furthermore, the local undergraduate teaching system relies heavily on memorization and testing memorized contents, rather than assessing the application of knowledge. This may reinforce in students the belief of needing to know all the facts, rather than admitting to having to refer to guidelines or other sources in clinical practice [58]. Both doctors and nurses in Palestine demonstrated equally little understanding of medical errors in a previous study, compared with the medical students in this study [25]. In Palestine, punitive response to medical error is prevalent and this influences local culture around dealing with medical errors, which is often felt to be the most difficult and oppressive aspect of patient safety culture for local healthcare professionals [33–35]. Other factors, possibly contributing to the negative culture around medical errors locally, could be a fear of publication of errors, leading to a negative impact on the reputation of the doctors and their private practice as well as a negative impact on their clinical evaluation and reputation.

Such conflicting understanding of medical error and its causes is also reflected in the domain of disclosure responsibility, where a large proportion of students found that all errors should be reported, but the majority felt that informing patients of errors would only need to be done if patients had come to harm. This can also be interpreted as the students realizing that if harm had occurred, a mistake would be obvious to the patient, but if no harm had occurred the patient might not be aware of it and, thus, alleviating the need for transparency in such situations. Moreover, disclosing errors to patients might negatively affect students’ image and lead to patients’ refusal for students to participate in their care. In the Palestinian healthcare system, healthcare professionals tend to be dominant over patients in clinical practice. They often feel that they know what is best for their patients [25, 34, 35]. A true decision sharing culture does not exist in clinical practice. This is partly due to limited choices open to patients [59], but is also reflected in the results of this study, where > 80% of participants thought that patients were unaware of the safety of the care provided to them. Similarly, medical students did not find that involving patients in their care was important; an item that had also been rated as less important by local healthcare professionals in a previous study [25]. In this point also, local culture is reflected within the medical students’ attitudes. Interestingly, male participants showed more positive attitudes in disclosure responsibility than female participants. In local reality, male students often present with more confidence than female students and this might simply be an expression of this culture of paternalism. Possibly, local male students feel less prone to make mistakes or they would be more confident in dealing with such mistakes and less critical of themselves than their female counterparts. This aspect of the role of gender would have to be more closely investigated in another study.

Students from University 2, who had received some structured patient safety training in their Year 4, showed a more positive understanding of error and error causation. They also demonstrated significantly more positive views on error reporting, despite the fact that their clinical training had been very similar and delivered by the same teachers in the same facilities. This difference between students from the two universities could be due to the curriculum contents on medical errors at University 2, which included a module on the opportunity errors pose for learning in individuals and organisations [14, 17, 55, 60, 61]. Hence, ‘theoretical learning’ of error causation could contribute to counteract the impact of the ‘hidden curriculum’. These findings, underline the need for formal delivery of patient safety contents within the undergraduate curriculum in conjunction with organisational change towards a non-punitive approach to enable medical students and healthcare professionals to learn from medical error [14, 55, 60, 61].

## Conclusion

The patient safety attitudes displayed by medical students in the Gaza Strip were slightly positive and showed surprising resemblance among students from both universities as well as with those among healthcare professionals of the same region. This underlines the power of the ‘hidden curriculum’, where students adjust to prevalent cultures in local hospitals, while they do their clinical training. Furthermore, it highlights the need for a systematic inclusion of patient safety contents in local undergraduate curricula.

## Data Availability

All data are available from the corresponding author on reasonable request.
